# Effects of Circadian Misalignment on Young and Aging Salivary Glands

**DOI:** 10.1177/00220345251372506

**Published:** 2025-10-18

**Authors:** H. Mortazavi, R. Said, G.S. Katselis, P. Chumala, G. Pannone, S. Papagerakis, P. Papagerakis

**Affiliations:** 1 Department of Anatomy, Physiology, and Pharmacology, College of Medicine, University of Saskatchewan, Saskatoon, SK, Canada; 2 Centre Hospitalier Universitaire de Québec Research Centre, Axe Maladies Infectieuses et Immunitaires, Université Laval, Québec, QC, Canada; 3 ARThrite Research Center (Arthrite Research Treatment), Centre Hospitalier Universitaire de Québec Research Centre, Université Laval, Québec, QC, Canada; 4 Interdisciplinary Center for Clinical and Applied Research and Entrepreneurship/Centre interdisciplinaire pour la recherche clinique et appliqué, Faculté de Médecine Dentaire, Université Laval, Québec, QC, Canada; 5 Department of Medicine, Canadian Centre for Rural and Agricultural Health, Mass Spectrometry Lab for Omics Research, College of Medicine, University of Saskatchewan, Saskatoon, SK, Canada; 6 Canadian Centre for Rural and Agricultural Health, Mass Spectrometry Lab for Omics Research, College of Medicine, University of Saskatchewan, Saskatoon, SK, Canada; 7 Department of Clinical and Experimental Medicine, University of Foggia, Foggia, Italy; 8 Department of Ophthalmology and ENT–Head and Neck Surgery, Faculty of Medicine, Université Laval, Québec, QC, Canada

**Keywords:** circadian rhythms, saliva, salivary physiology, Sjögren’s disease, autoimmunity, cytokines

## Abstract

Several empirical observations strongly suggest that salivary function is regulated by the circadian clock. Salivary volume, electrolytes levels, and saliva protein composition all show 24-h cycle fluctuations. The exact effects of circadian disruption on salivary gland (SG) physiology and its potential role in salivary pathologies have not been elucidated. Here, we examined the effects of circadian disruption on SG structure, functional gene and protein expression, and immune status using several circadian knockout (KO) mice models where we targeted the following canonical clock genes: brain and muscle ARNT-like 1 protein (*Bmal1*) KO, Period2 (*Per2*) KO, Cryptochrome1 (*Cry1*) KO, Cryptochrome2 (*Cry2*) KO, and Cryptochrome 1 and 2 double KO (*DKOCry*). All mice were females of young and old age, and data were compared with wild type (control) mice. Our results showed that circadian dysregulation had limited effects on stimulated salivary secretion and SG structure. Yet, circadian disruption significantly affected the expression of several key salivary markers, including mucins, amylase, and aquaporins, in young and aged SGs with increased amylase and acidic mucin production observed in several KO models. In addition, targeting of clock genes has resulted in subtle alterations of the salivary immune microenvironment with increased lymphocyte infiltration and upregulated levels of proinflammatory cytokines. These immune shifts were more pronounced in aged glands with the most proinflammatory phenotypes observed in *DKOCry* and *Bmal1KO* mice. Collectively, our results implicate the circadian clock in the intricate temporal regulation governing SG function. Our data also suggest that circadian dysregulation may predispose to increased tissue stress and inflammation. Exploration of salivary system chronobiology represents a new avenue for salivary disease prevention and treatment.

## Background

The circadian clock, a natural 24-h internal system found in most organisms, governs almost all physiologic processes, enabling adaptation to environmental changes (Adeola et al. 2019). In mammals, this system is centered in the suprachiasmatic nucleus, which oversees subsidiary clocks in various brain regions and organs (Reppert and Weaver 2002). At the molecular level, this system is maintained and controlled by a diverse group of transcription factors expressed by clock genes (Papagerakis et al. 2022). The core clock genes include brain and muscle ARNT-like 1 protein (*BMAL1*), circadian locomotor output cycles kaput protein (*CLOCK*), periods (*PER1*/*PER2*), and cryptochromes (*CRY1*/*CRY2*), among others (Said et al. 2020). Transcription-translation feedback loops involving these genes drive the circadian rhythm (Zheng et al. 2014). It is estimated that 43% of all protein-coding genes exhibit circadian oscillations in transcription (Zhang et al. 2014). Disruptions in the circadian clock have been linked to various physiologic and pathologic conditions affecting varied health outcomes, including metabolic disorder, cancer susceptibility, and development of autoimmunity (Adeola et al. 2019).

In the context of saliva, several observations strongly suggest that salivary function is regulated by the circadian clock. Multiple reports showed distinct circadian variations in saliva secretion with clear diurnal fluctuations in salivary volume, electrolytes, and protein composition (Dawes 1972; Dawes and Ong 1973; Zheng et al. 2012). There is a clear diurnal variation in salivary immunoglobulin A levels (peaking through the night), which is thought to be mediated by suprachiasmatic nucleus stimulation of the salivary sympathetic nerves (Wada et al. 2017). We and others have highlighted the role of the molecular circadian clock in influencing aquaporin 5 (Aqp5) expression, a key player in regulating water permeability and salivary fluid secretion, where we showed that Aqp5’s expression is influenced by *BMAL1*-*CLOCK* heterodimer and that its expression levels exhibit rhythmic patterns, especially in the submandibular gland (Zheng et al. 2012; Satou et al. 2019). However, the exact effects of circadian dysregulation on salivary function and health are still greatly understudied with several unknowns, with no study in the literature having hitherto thoroughly investigated the impact of molecular circadian disruption on salivary function.

This study aims to address this gap by performing a comprehensive analysis of salivary functional gene and protein expression, immune status, glandular structure, and saliva production in 5 mouse knockout (KO) models targeting the following core clock genes: brain and muscle ARNT-like 1 protein (*Bmal1*) KO, Period2 (*Per2*) KO, Cryptochrome1 (*Cry1*) KO, Cryptochrome2 (*Cry2*) KO, and Cryptochrome 1 and 2 double KO (*DKOCry*).

## Materials and Methods

### Mice

This study was approved by the University of Saskatchewan’s Animal Research Ethics Board and adhered to the guidelines set forth by the Canadian Council on Animal Care for humane animal use (protocol 20200010; the approved protocol included the research question, key design features, and analysis plan). We have complied with the ARRIVE 2.0 guidelines (Animal Research: Reporting In Vivo Experiments) for our animal protocol and experimentation (a full checklist is provided in the Appendix). Exclusively female mice aged between 12 and 29 wk were utilized due to the higher prevalence of immune-based salivary gland (SG) pathologies such as Sjögren’s syndrome (SS) in females (Qin et al. 2015). Mice were analyzed from 5 clock gene KO colonies and 1 wild type (WT) colony (serving as control) and categorized as young (12 wk) and middle-aged or aging (29 wk). A total of 48 mice were used (8 mice per genotype group; 4 young and 4 aging). Genotyping protocol and primer sequences are detailed in the Appendix.

### Sample Collection

Collections of saliva and blood were scheduled at 2 distinct times: 9:00 to 10:00 a.m. (rest period) and 9:00 to 10:00 p.m. (active period). SGs were harvested, including the submandibular, parotid, and sublingual glands (SLGs). Collection procedures are detailed in the Appendix.

### Histologic Staining and Analysis

We utilized hematoxylin and eosin staining, as well as periodic acid–Schiff and alcian blue staining (PAS/AB), to characterize SG structure and morphology (Appendix).

### Immunostaining

Anti-CD3 antibody (14-0031-82; Thermo Fisher), anti-amylase (15845-1-AP; Thermo Fisher), and anti-AQP5 (A5-99403; Invitrogen) were used (Appendix).

### Quantitative Real-time Polymerase Chain Reaction

We analyzed the gene expression levels of interleukin 17 alpha (*IL17A*), SS A antigen (*SSA*/Ro60), tumor necrosis factor alpha (*TNFa*), *Aqp5*, amylase (*Amy*), and mucin 19 (*Muc19*). Beta-2 microglobulin (*B2M*) was selected as a reference gene, as it is unaffected by circadian regulation (Appendix).

### Quantitative Proteomics Analysis of Mouse Blood Samples

Blood samples from aging mice across all colonies were utilized for the assessment of protein quantities (Appendix).

### Statistical Analysis

All statistical analyses were performed by R Studio and SPSS version 29 (Appendix).

## Results

### Circadian Disruption Effects on Stimulated Saliva Secretion and SG Size

Examination of stimulated salivary secretion uncovered limited circadian-dependent differences after normalizing the saliva levels to body weight. Young *Per2KO* mice Mean ± SD (5.1538 ± 0.8696 µL/g, *P* = .041) produced significantly more saliva in the morning as compared with WT, while old *Cry1KO* mice Mean ± SD (1.5504 ± 0.2547 µL/g, *P* = .009) had reduced nighttime saliva output (Fig. 1A, B). Gross inspection of the submandibular SGs revealed no significant differences in gland weight among the colonies at 29 wk. In the 12-wk groups, the *Bmal1KO* glands were noticeably lighter when compared with WT, *Per2KO*, and *CryDKO* (*P* = 0.0922, 0.1101, and 0.0692, respectively; Fig. 1C) with some significant differences in gland area between young clock KO mice and WT controls (Fig. 1D).

**Figure 1.**
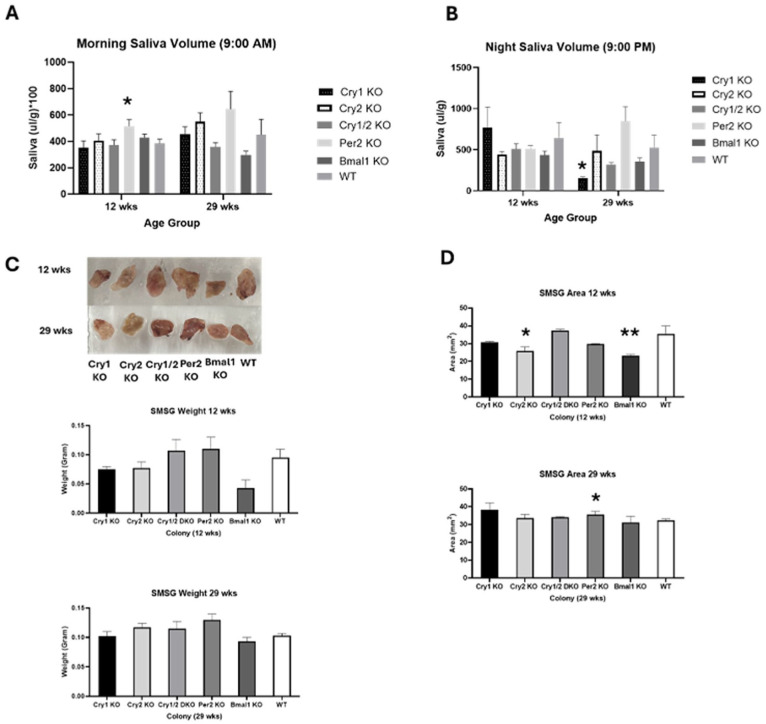
The impact of clock gene knockouts (KOs) on stimulated saliva production and gland size. (**A**, **B**) Stimulated mean saliva production, normalized by weight, in 12- and 29-wk-old clock KO mouse colonies as compared with wild types (WTs). Saliva production was measured in the morning (9:00 a.m.) and at night (9:00 p.m.). When compared with WTs, among 12-wk-old clock KO mouse colonies, *Per2KO* mice display increased saliva production in the morning, while among the 29-wk-olds, *Cry1KO* mice show reduced saliva production at night. (**C**) Upper panel: Representative images of the excised hemisubmandibular salivary glands in different strains at 12 and 29 wk. Lower panel: Average hemisubmandibular gland weight measured in grams across the different strains; no significant differences were noted in gland weight. However, in the 12-wk groups, the *Bmal1KO* glands were noticeably lighter when compared with WT, *Per2KO*, and *DKOCry* (*P* = 0.0922, 0.1101, and 0.0692, respectively). (**D**) Average mandibular gland area size measured in square millimeters across the different strains. At 12 wk of age, the *DKOCry* strain exhibits the largest mandibular gland area, followed closely by the WTs. *Cry2KO* and *Bmal1KO* mice exhibited significantly smaller mandibular gland areas as compared with WT. Among older mice, only *Per2KO* glands are significantly smaller when compared with their WT counterparts. *P ≤ 0.05 and **P ≤ 0.001 vs WT. *n* = 3 or 4 per genotype. Data are presented as Mean ± SEM. SMSG, submandibular salivary gland.

### Targeting the Core Clock Genes Resulted in Limited Effects on SG Structure

Analysis of hematoxylin and eosin–stained images did not reveal any major microstructural changes in acinar or ductal cells except for a higher rate of acinar epithelial reduction observed in *DKOCry* mice (Fig. 2A, B). Focal lymphocytic sialadenitis was assessed by measuring the focus score (mononuclear aggregates per 4 mm^2^ of glandular area, *n* = 4 per genotypes; Fig. 2C); generally, our focus scores show that targeting the clock genes, particularly *Crys* and *Bmal1*, resulted in mild lymphocytic infiltration that usually does not reach the threshold for pathologic focal lymphocytic sialadenitis. With regard to salivary tissue architecture, our analyses revealed that aging glands show smaller acini accompanied by histologic aspects of atrophy with reduced parenchymal/stromal ratio when compared with their younger counterparts in all of the colonies (Fig. 2D, E). These effects were most pronounced in the *Per2KO* mice, as older mice showed significantly smaller acini and increased stromal fibrosis when compared with their younger counterparts. No significant differences in acini size and parenchymal/stromal ratio were noted between the clock KO genotypes.

**Figure 2.**
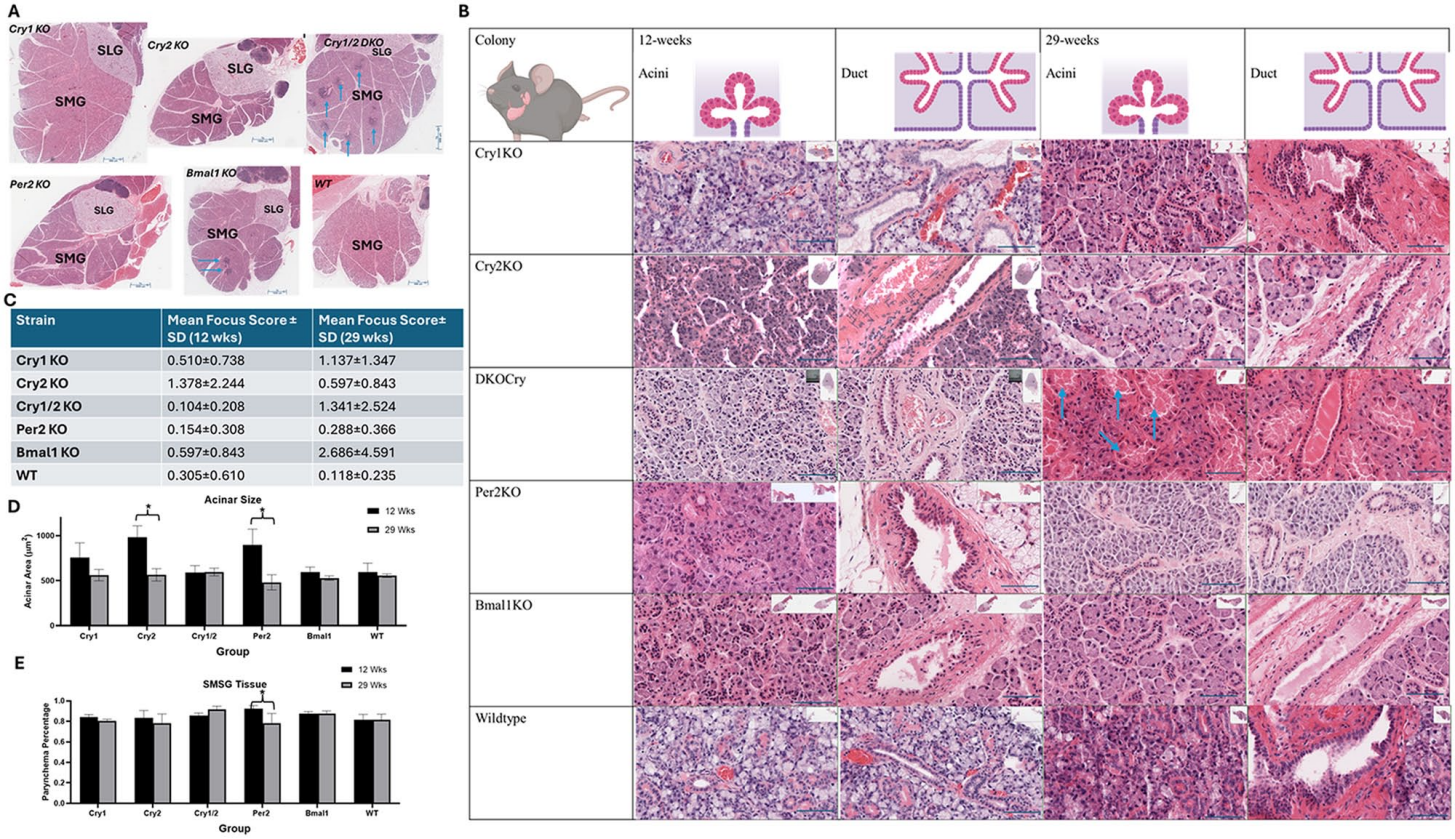
The impact of circadian disruption and aging on salivary gland structure. (**A**) Representative hematoxylin and eosin–stained sections of the aged salivary gland (29 wk) from each colony. Observed intraglandular lymphocytic foci in aging *DKOCry* and *Bmal1KO* (blue arrows). SLG, sublingual gland; SMG, submandibular gland. Scale bars: stated. (**B**) Zoomed-in inspection did not reveal any major microstructural changes in acinar or ductal cells except for a higher rate of acinar epithelial reduction in aging *DKOCry* mice (blue arrows). Scale bar: 100 μm in all panels. (**C**) Focal lymphocytic sialadenitis was assessed by measuring the focus score (mononuclear aggregates per 4 mm^2^ of glandular area, *n* = 4 per genotype). The focus score was >1 in four 12-wk samples of the examined 24 (1 WT, 1 *Cry1KO*, 1 *Cry2KO*, and 1 *Bmal1KO*) and in six 29-wk samples of the examined 24 (2 *Cry1KO*, 1 *Cry2KO*, 1 *DKOCry*, and 2 *Bmal1KO*). Generally, the scores show that targeting of the clock genes, particularly *Cry* and *Bmal1*, resulted in mild lymphocytic infiltration that usually does not reach the threshold for pathologic focal lymphocytic sialadenitis. (**D**) Average acini sizes are compared across different clock knockout colonies at the 2 age groups. Most colonies show a noticeable decline in acini size from 12 to 29 wk, with *Cry2KO* and *Per2KO* showing significant decreases in acini size with age, resulting in glandular atrophy. *P ≤ 0.05. *n* = 4 per genotype. (**E**) Average parenchymal/stromal ratios for the same colonies and age groups. The stromal portion of the gland appears to generally increase with age across the colonies. The aging *Per2KO* group shows the most pronounced atrophy and fibrosis, as evaluated by a reduction in parenchymal/stromal ratio with age. *P ≤ 0.05. *n* = 4 per genotype. Data are presented as Mean ± SEM. KO, knockout; SMSG, submandibular salivary gland; WT, wild type.

### Targeting the Core Clock Genes Differentially Affected Salivary Functional Markers

Our PAS/AB staining and *Muc19* gene expression show a substantial increase in acidic mucin production in the SLGs of *Per2KO*, *Bmal1KO*, and *CryDKO* mice when compared with WTs (Fig. 3A–G). Similarly, the 3 colonies showed augmented levels of *Amy* gene and protein expression in their parotid glands when compared with WTs (Fig. 4A, B). As for *Aqp5*, only targeting *Per2* resulted in a significant reduction of *Aqp5* gene and protein expression levels in aging glands (Fig. 4C–E).

**Figure 3.**
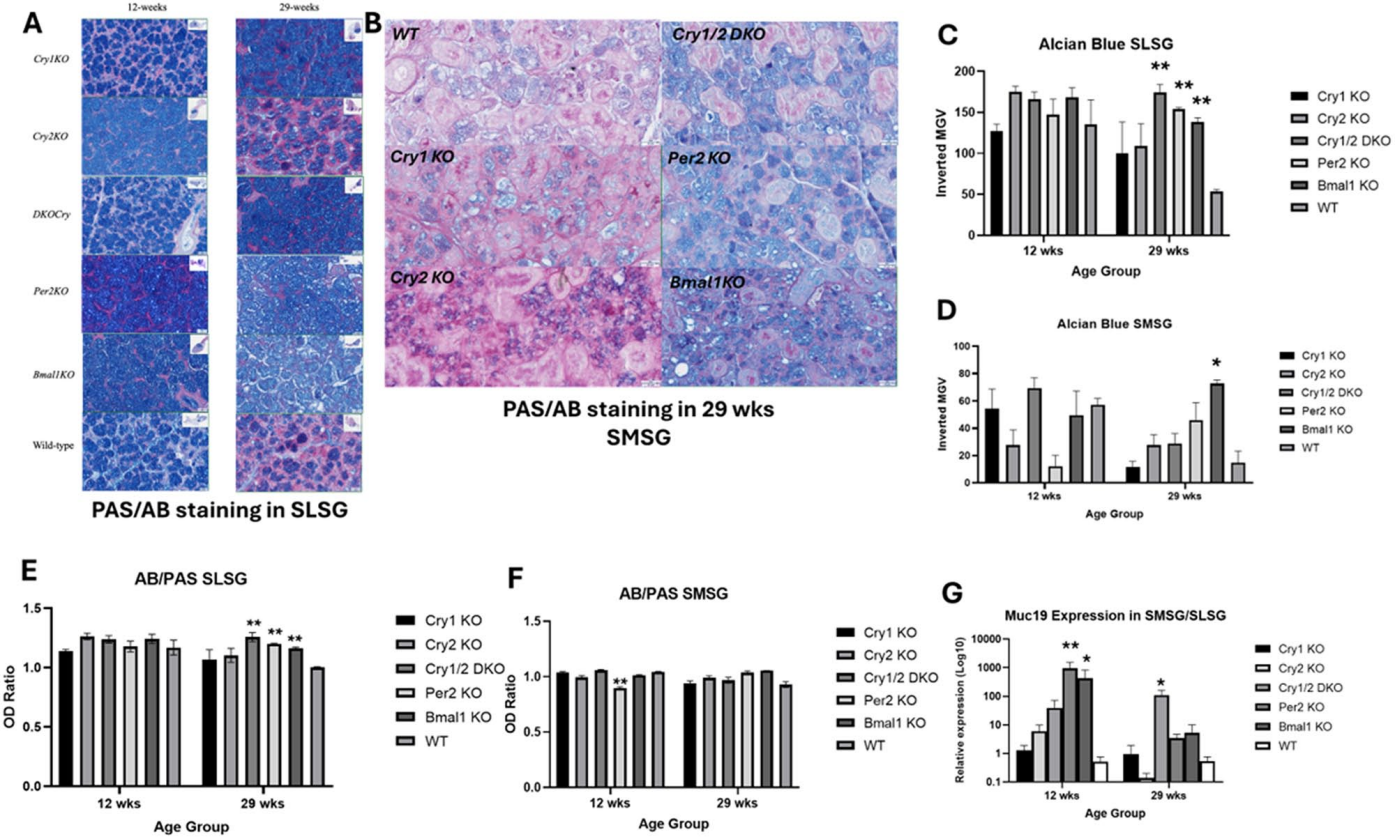
The impact of circadian disruption on salivary mucin production. (**A**) Representative microscopic images show periodic acid–Schiff and alcian blue (PAS/AB) staining in the sublingual gland. (**B**) Representative microscopic images show PAS/AB staining in the submandibular gland (29 wk). Scale bars: 25 μm for panels A and B. (**C**, **D**) AB intensity quantification analysis (represented as inverted mean gray values [MGVs]) revealed a significant increase in acidic mucin production in 29-wk *Cry1/2*, *Per2*, and *Bmal1KO* SLSG and *Bmal1KO* SMSG as compared with their WT littermates. *P ≤ 0.05 and **P ≤ 0.001 vs WT. *n* = 3 to 5 female mice per group. (**E**, **F**) AB/PAS optical density (OD) ratio revealed a significant shift toward acidic mucin production in the 29-wk *Cry1/2KO*, *Per2KO*, and *Bmal1KO* SLSG; such shifts usually occur in tissue stress and inflammation. *P ≤ 0.05 and **P ≤ 0.001 vs WT. *n* = 3 to 5 female mice per group. (**G**) Relative mRNA levels of mucin 19 (*Muc19*) in samples collected from the submandibular and sublingual glands. *P ≤ 0.05 and **P ≤ 0.001 vs WT. *n* = 4 female mice per group. Data are presented as mean ± SEM. KO, knockout; SLSG, sublingual salivary gland; SMSG, submandibular salivary gland; WT, wild type.

**Figure 4.**
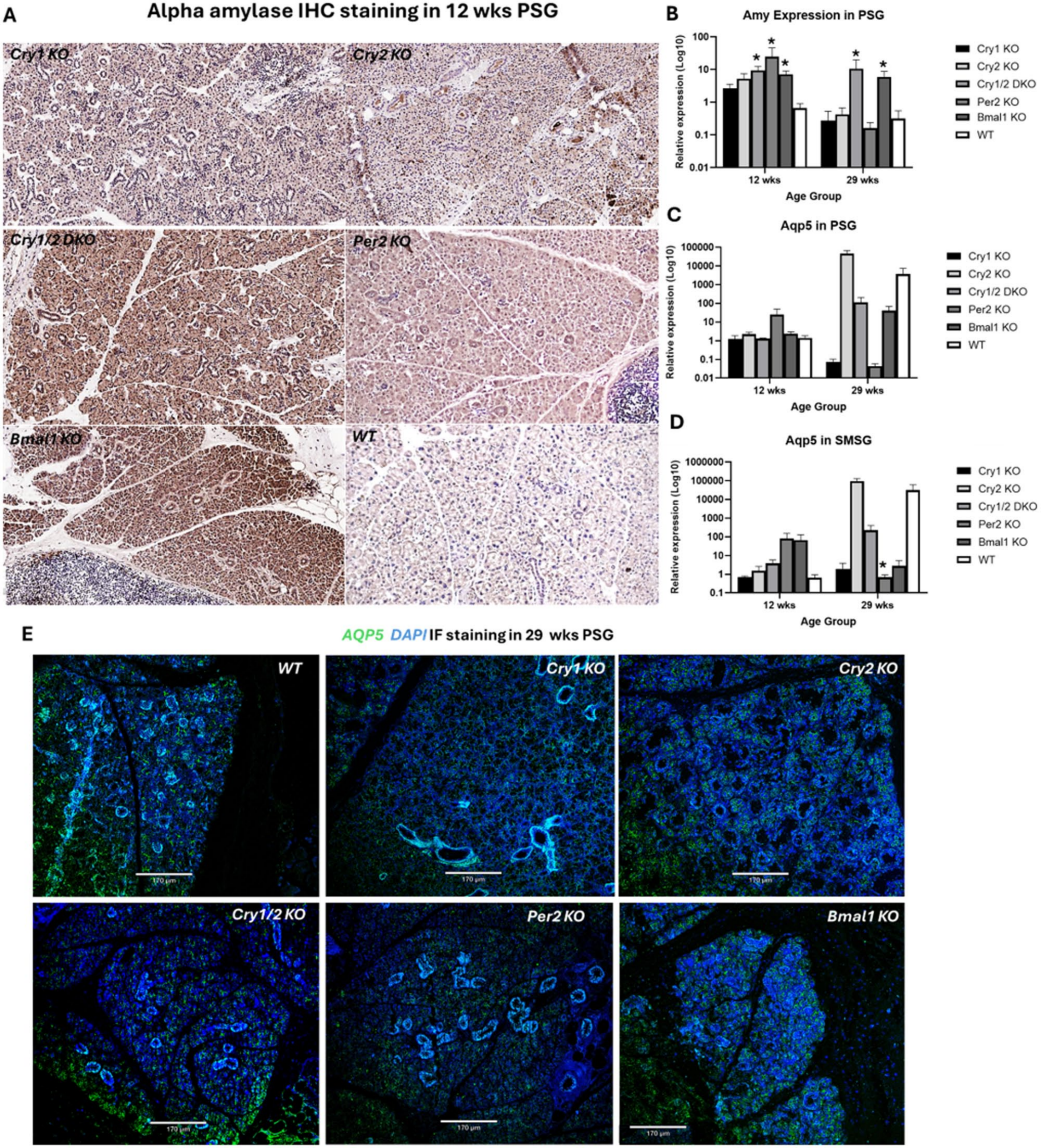
The impact of circadian disruption on salivary functional markers. (**A**) Representative sections of the 12-wk parotid salivary gland (PSG) from each colony after salivary alpha-amylase immunostaining. Increased amylase immunoreactivity was noted in all circadian knockouts (KOs) as compared with wild type (WT), particularly in *Bmal1KO* and *Cry1/2KO*. Scale bars: 100 μm in all panels. (**B-D**) Gene expression analysis of amylase (*Amy*) and aquaporin 5 (*Aqp5*). *P ≤ 0.05 and **P ≤ 0.001 vs WT. *n* = 4 per genotype. Relative mRNA levels: (B) *Amy* in samples collected from the PSG, (C) *Aqp5* in samples collected from the PSG, and (D) *Aqp5* in samples collected from the submandibular salivary gland (SMSG). *Per2KO* mice show age-specific effects on amylase and *Aqp5*, with higher levels in young mice and lower levels in older mice. No significant changes in amylase or *Aqp5* expression were observed in *Cry1KO* or *Cry2KO* mice. (**E**) Representative sections of the 29-wk PSG from each colony after AQP5 immunostaining. No clear differences were noted among the groups except for a slightly weaker signal in *Per2KO* and *Cry1KO*. Scale bars: 170 μm in all panels. Data are presented as mean ± SEM.

**Figure 5.**
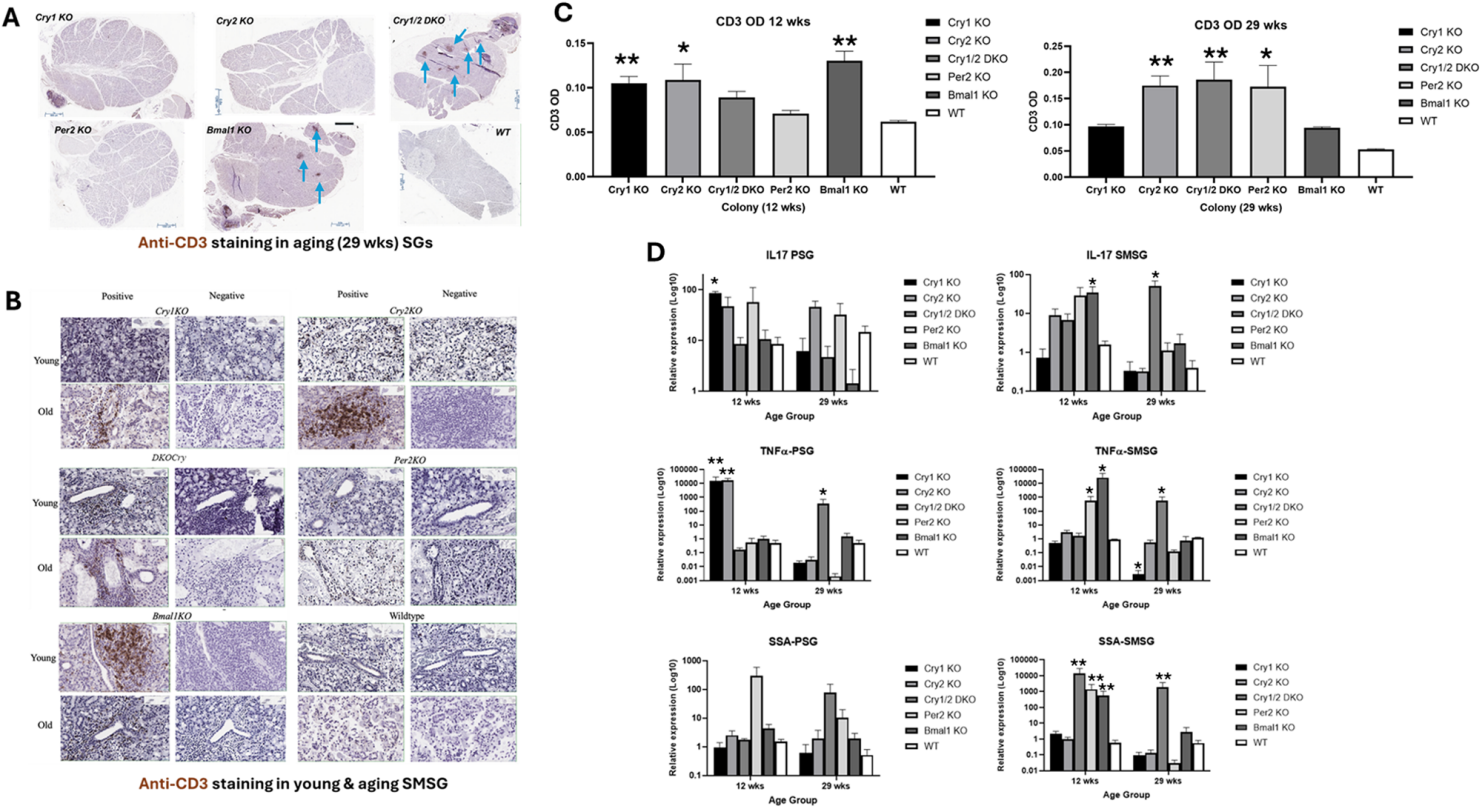
Targeting the different components of the molecular clock effects on immune markers in salivary glands (SGs). (**A**) Representative sections of the aged salivary gland (29 wk) from each colony after CD3 immunostaining, illustrating T-cell distribution. An increase in T-cell–rich intraglandular lymphocytic foci was noted in the aging *DKOCry* and *Bmal1KO* groups when compared with the others (blue arrows). Scale bars: stated. (**B**) Representative zoomed-in images of young and aging sections show increased periductal lymphocytic infiltration in the old *Cry1KO*, *CryDKO*, and *Bmal1KO* groups. Scale bars: 25 μm in each panel. (**C**) Semiquantitative analysis of CD3 optical density showed a significantly higher rate of T-lymphocyte infiltration in different young and aging circadian knockout (KO) colonies when compared with their wild type (WT) counterparts. The colonies with the highest CD3 levels were the young *Bmal1KO* and aging *CryDKO.* *P ≤ 0.05 and **P ≤ 0.001 vs WT. *n* = 4 per genotype. (**D**) Relative mRNA levels of 3 immune markers (TNFα, IL-17, and SSA antigen) in the parotid salivary gland (PSG) and submandibular salivary gland (SMSG). Targeting both *Cry* genes has resulted in significantly higher levels of TNFα, IL-17, and SSA antigen in the aging SMSG. *P ≤ 0.05 and **P ≤ 0.001 vs WT. *n* = 4 per genotype. Data are presented as mean ± SEM.

### Targeting Cryptochrome Genes Resulted in Proinflammatory Salivary Features

Our T-cell abundance analysis showed enhanced infiltration of T lymphocytes in the young and aging circadian KO colonies when compared with their WT counterparts with a more pronounced infiltration observed in aging glands (Fig. 5A, B). The colonies with the highest CD3 levels were the young *Bmal1KO* and aging *CryDKO* (Fig. 5C). With regard to the cytokine profile, targeting both *Cry* genes has resulted in significantly higher levels of *TNFα*, *IL-17*, and *SSA* antigen in the submandibular glands (Fig. 5D). Serum proteomics analysis of the aging mice profiling unveiled several altered pathways reflective of immune responses in the different KO models (Appendix Table 2, Appendix).

## Discussion

In this study, we unveiled the multifaceted effects of circadian dysregulation on saliva production, glandular structure, functional gene expression, and salivary immune environment.

It is well established that salivary flow follows a distinct circadian rhythm with notable daily fluctuations in salivary flow rate and composition. These rhythms are important for effective nutrition and self-defense functions of saliva, with the flow rate typically increasing during the active phase and decreasing at the rest phase (Nater et al. 2007). Previous studies showed that unstimulated saliva secretion follows circadian rhythms in flow rate and protein, sodium, potassium, calcium, and chloride concentrations in humans (Dawes 1972; Dawes and Ong 1973). However, the impact of dysregulation of the circadian rhythm on salivary secretion has never been investigated. Our data show that targeting different clock genes did not result in clear significant changes in the volume of stimulated saliva collected after parasympathetic stimulation. The lack of significant effects on stimulated salivary flow indicates that targeting the circadian clock does not significantly influence autonomic parasympathetic regulation of salivary function.

Aging SGs in the circadian KO colonies showed increased stromal proliferation (fibrosis), reduced parenchymal tissues, and smaller average acinar size (atrophy) when compared with their younger counterparts. Young *Per2KO* mice had a smaller mandibular gland with low connective tissue (i.e., high parenchyma/stroma ratio) and larger acini when compared with their WT littermates, while older *Per2KO* mice demonstrated the exact reverse phenotype. The effects of the circadian rhythms on gland size and structure have been reported in other endocrine and exocrine glandular tissues (Ulrich-Lai et al. 2006; Kalsbeek et al. 2012; Bedrosian et al. 2016; Lee et al. 2022). For example, it was shown that chronic disruption of circadian rhythms (e.g., shift work and exposure to constant light) can lead to long-term changes in adrenal gland size accompanied by alterations in hormone release and feedback mechanisms (Ulrich-Lai et al. 2006). Our hematoxylin and eosin staining did not reveal any major microstructural changes in acinar or ductal cells except for a higher rate of cellular degeneration observed in *DKOCry*, suggesting that disrupting the circadian clock does not significantly affect SG development.

Our salivary functional analyses implicate the circadian clock in the temporal regulation of the 3 key SG functions: enzymatic digestion, mucus production, and water transport. Indeed, our quantitative real-time polymerase chain reaction and immunohistochemistry analyses revealed that *Bmal1KO* and *DKOCry* mice expressed significantly higher levels of *Amy* when compared with WT in young and aging mice. For the *Per2KO* mice, the effects appear to be age specific, with significantly higher levels of *Amy* in younger glands and lower levels in aging glands when compared with WTs. The circadian nature of amylase secretion is very well documented and often aligns with the anticipated time of food intake that is driven by clock gene activity (Nater et al. 2007; Out et al. 2013). The increased amylase production noted in our mice suggests an increased sympathetic drive in these colonies, a phenomenon that is observed in individuals with circadian disruption. Indeed, previous studies have shown that shift work or jet lag can cause increased sympathetic nervous system activation with increased serum norepinephrine levels (Aziz et al. 2021; Wang et al. 2021). The impact of the circadian disruption on amylase was also reported in older adults and attributed to the increased sympathetic drive observed with increasing age and reduced physical activity (Strahler et al. 2010).

Acidic mucin production was significantly upregulated in the aging *Bmal1KO*, *Per2KO*, and *DKOCry* mice. Indeed, our gene expression level analysis of *Muc19* (the major gel-forming mucin in the saliva of mice, particularly in SLG) and PAS/AB staining revealed a higher mucin level in the SLG of the 3 KOs when compared with WTs. Unlike amylase, there is limited specific information about the circadian rhythm of salivary mucin expression, and this is the first study showing that salivary mucin production is influenced by the clock genes. Increased production of acidic mucins in the SGs is commonly observed during periods of tissue stress, inflammation, or infection. For example, in CCL28-deficient mice, which have impaired mucosal immunity, acidic mucin production is reduced, and this is associated with increased bacterial invasion (Kaibori et al. 2024). Furthermore, proinflammatory cytokines (e.g., *TNF-α* and *IL-17*, which are increased in these colonies) are known to stimulate mucin synthesis and can shift mucin production toward more acidic forms as part of the tissue’s reaction to immune activation and injury (Kaibori et al. 2024). Based on the aforementioned, the observed increase in SLG acidic mucins in the circadian KOs likely reflects a direct effect of clock gene loss on mucin gene regulation and an indirect effect via increased inflammatory signaling and tissue stress.

Our data suggest that the *Aqp5* gene is a downstream target of PER2 as *Per2KO* showed marked decrease in *Aqp5* in aging parotid and submandibular SGs. AQP5 water channels provide a major route for osmotically driven water movement in acinar and intercalated ductal cells (Flodby et al. 2017). Our group first demonstrated that the expression levels of *Aqp5* showed regular oscillatory patterns under light/dark and complete-dark conditions and *Bmal1* overexpression resulted in increased *Aqp5* expression levels (Zheng et al. 2012). In a subsequent study, Satou et al. (2019) also observed a circadian rhythmic pattern in *Aqp5* mRNA expression in rat whole SGs. Interestingly, the *Aqp5* rhythmic expression patterns were synchronous with *Per2* expression, which further suggests that *Per2* acts as a direct regulator of AQP5 (Satou et al. 2019). Conversely, Uchida et al. (2018) suggested that the daily rhythm of *Aqp5* expression is predominantly controlled by the central clock in the suprachiasmatic nucleus rather than by the autonomous salivary molecular clock, suggesting that more studies are needed to conclude how the clock exactly regulates *Aqp5* expression. No significant changes in amylase, mucin, or aquaporin expression were noted in *Cry1KO* and *Cry2KO* mice, suggesting that elongating or shortening the circadian period (noted in people with morning and night preference chronotypes, respectively) does not appear to significantly alter saliva enzymatic, mucinous, and/or watery composition.

With regard to the immune markers and features in SGs after circadian disruption, we found that all our circadian KO colonies showed a higher degree of T-cell infiltration when compared with WTs. The increase was more pronounced in aging glands, and the colonies with the highest lymphocytic infiltration were the *DKOCry* (in the aging group) and the *Bmal1KO* (in the young group). The chronic phlogistic infiltrates were mostly concentrated surrounding interlobular collecting and intralobular striated ducts. The circadian clock plays a substantial role in regulating lymphocyte trafficking and infiltration as lymphocyte migration occurs in a circadian manner (Druzd et al. 2017). Similar to our observed immune phenotype in the aging *DKOCry* SGs, Cao et al. (2017) reported increased infiltration of leukocytes in adult *CryDKO* mice lungs and kidneys. Expression of *IL-17* and *TNF-α* was significantly upregulated in the aging *DKOCry* SGs. The circadian clock plays a crucial role in regulating the expression of *IL-17* and *TNF-α*. Indeed, core clock genes such as retinoic acid receptor–related orphan receptor alpha (*Rorα*) and nuclear receptor subfamily 1 group D member 1 (*NR1D1*) were found to induce the expression of IL-17A and IL-17F in Th17 cells (Amir et al. 2018). The interaction between TNF-α and the circadian clock appears to be bidirectional with each influencing the other’s function and expression (Cermakian et al. 2014). It was previously reported that the absence of *Cry1* and *Cry2* leads to increased levels of serum TNF-α (Cavadini et al. 2007; Cermakian et al. 2014). Our results further consolidate the *Cry*-mediated regulation of TNF-α as targeting either *Cry1* or *Cry2*, individually or together, resulted in significant upregulation of *TNF-α* mRNA levels. We also assessed the expression levels of the SSA antigen. Anti-SSA/Ro antibodies are found in approximately 50% to 70% of patients with SS, and their presence is among the classification criteria of the American College of Rheumatology and European Alliance of Associations for Rheumatology for primary SS (Baldini 2022). Similar to *IL-17* and *TNF- α*, the highest upregulation in SSA antigen levels was noted in the aging *DKOCry* SGs. However, further assessment is still needed to comprehensively evaluate any SS-like pathologic changes. This includes measuring anti-SSA/Ro antibodies in serum samples (which could not be assessed reliably here due to serum sample volume constraints) as tissue expression alone does not fully capture autoimmune status. Additionally, performing flow cytometry or immunostaining on SG tissues is essential to clearly identify and quantify the infiltrating immune cell populations, such as the different T-cell subsets, B cells, germinal centers (BCL2 and 6), and other immune cells (e.g., CD21-positive follicular dendritic cells). In a recent study, Liu et al. (2022) demonstrated the potential links between SS and the clock as they showed that *Cry1*, *Cry2*, and *Bmal1* gene expression was significantly reduced in the SGs of patients and NOD mice. In addition, they showed that melatonin treatment in NOD mouse models nearly restored clock gene activity in SGs, reduced immune cell infiltration, and decreased proinflammatory responses (Liu et al. 2022).

Each circadian KO model used in this study carries distinct systemic phenotypes, which must be considered when interpreting the observed changes in the SGs. *Cry1KO* and *Cry2KO* mice display altered circadian periodicity but generally retain rhythmicity: *Cry1KO* mice have a shortened circadian period, while *Cry2KO* mice have a lengthened period. Both are viable and fertile and exhibit only mild metabolic or behavioral changes under standard conditions (van der Horst et al. 1999). In contrast, *DKOCry* and *Bmal1KO* mice are completely arrhythmic in constant darkness, with profound systemic effects. *DKOCry* mice are viable but display metabolic syndrome features, immune dysregulation, and increased tumor susceptibility (van der Horst et al. 1999). *Bmal1KO* mice exhibit the most severe systemic phenotype, including complete loss of circadian rhythmicity, premature aging, infertility, metabolic syndrome, and reduced life span (Bunger et al. 2000). *Per2KO* mice typically show a shortened free-running period, but many—especially females—become arrhythmic after prolonged constant darkness, with increased cancer risk and metabolic disturbances (Bae et al. 2001). The phenotypes that we observed in the SGs of these models most likely reflect an interplay of local (gland-intrinsic) circadian disruption and systemic consequences of global clock gene loss. Our findings of altered expression of the salivary markers (i.e., the reduction of Aqp5 in *Per2KO* glands and increased acidic mucins and amylase) support a direct local role for clock genes in SG homeostasis. However, the immune alterations observed (increased T-cell infiltration and elevated IL-17 and TNF-α) are likely amplified by systemic immune dysregulation characteristic of global circadian disruption. Indeed, *DKOCry* and *Bmal1KO* mice are known to develop systemic immune abnormalities, including heightened autoimmunity and altered cytokine profiles, which can predispose to or exacerbate local glandular inflammation. The observed increase in lymphocytic infiltration and proinflammatory gene expression in these models’ SGs is consistent with this systemic immune activation.

In conclusion, our comprehensive investigation has unveiled a complex interplay between circadian rhythms and salivary function, with potential implications for SG immune regulation. While the specific mechanisms may vary among species, the fundamental role of the circadian clock in regulating physiologic processes is conserved across mammals, including humans. Therefore, it is plausible that similar disruptions in circadian rhythms could affect human SG health and immune function. Our findings open new avenues for understanding SG physiology and for the identification of clock genes as key regulators of SGs, rendering them an excellent potential therapeutic target for chronotherapy in salivary disorders.

## Author Contributions

H. Mortazavi, R. Said, contributed to conception and design, data acquisition, analysis, and interpretation, drafted and critically revised the manuscript; G.S. Katselis, P. Chumala, contributed to data acquisition, critically revised the manuscript; G. Pannone, contributed to data interpretation, critically revised the manuscript; S. Papagerakis, P. Papagerakis, contributed to conception and design, data interpretation, drafted and critically revised the manuscript. All authors gave their final approval and agree to be accountable for all aspects of the work.

## Supplemental Material

sj-docx-1-jdr-10.1177_00220345251372506 – Supplemental material for Effects of Circadian Misalignment on Young and Aging Salivary Glands Supplemental material, sj-docx-1-jdr-10.1177_00220345251372506 for Effects of Circadian Misalignment on Young and Aging Salivary Glands by H. Mortazavi, R. Said, G.S. Katselis, P. Chumala, G. Pannone, S. Papagerakis and P. Papagerakis in Journal of Dental Research

## References

[bibr1-00220345251372506] Adeola HA Papagerakis P Papagerakis S. 2019. System biology approaches and precision oral health: a circadian clock perspective. Front Physiol. 10:399. doi:10.3389/fphys.2019.00399 31040792 PMC6476986

[bibr2-00220345251372506] Amir M Chaudhari S Wang R Campbell S Mosure SA Chopp LB Lu Q Shang J Pelletier OB He Y. 2018. REV-ERBα regulates TH17 cell development and autoimmunity. Cell Rep. 25(13):3733–3749. 30590045 10.1016/j.celrep.2018.11.101 PMC6400287

[bibr3-00220345251372506] Aziz IS McMahon AM Friedman D Rabinovich-Nikitin I Kirshenbaum LA Martino TA . 2021. Circadian influence on inflammatory response during cardiovascular disease. Curr Opin Pharmacol. 57:60–70. 33340915 10.1016/j.coph.2020.11.007

[bibr4-00220345251372506] Bae K Jin X Maywood ES Hastings MH Reppert SM Weaver DR . 2001. Differential functions of mPer1, mPer2, and mPer3 in the SCN circadian clock. Neuron. 30(2):525–536. 11395012 10.1016/s0896-6273(01)00302-6

[bibr5-00220345251372506] Baldini C . 2022. Classification criteria in Sjögren’s syndrome. In: Bruyn GAW , editor. Sjögren’s syndrome and the salivary glands: novel techniques in diagnosis, management and treatment. Contemporary rheumatology. Cham (Switzerland): Springer. p. 29–35.

[bibr6-00220345251372506] Bedrosian TA Fonken LK Nelson RJ . 2016. Endocrine effects of circadian disruption. Annu Rev Physiol. 78:109–131. 26208951 10.1146/annurev-physiol-021115-105102

[bibr7-00220345251372506] Bunger MK Wilsbacher LD Moran SM Clendenin C Radcliffe LA Hogenesch JB Simon MC Takahashi JS Bradfield CA . 2000. Mop3 is an essential component of the master circadian pacemaker in mammals. Cell. 103(7):1009–1017. 11163178 10.1016/s0092-8674(00)00205-1 PMC3779439

[bibr8-00220345251372506] Cao Q Zhao X Bai J Gery S Sun H Lin D-C Chen Q Chen Z Mack L Yang H. 2017. Circadian clock cryptochrome proteins regulate autoimmunity. Proc Natl Acad Sci U S A. 114(47):12548–12553. 29109286 10.1073/pnas.1619119114 PMC5703267

[bibr9-00220345251372506] Cavadini G Petrzilka S Kohler P Jud C Tobler I Birchler T Fontana A. 2007. TNF-α suppresses the expression of clock genes by interfering with E-box–mediated transcription. Proc Natl Acad Sci U S A. 104(31):12843–12848. 17646651 10.1073/pnas.0701466104 PMC1937554

[bibr10-00220345251372506] Cermakian N Westfall S Kiessling S. 2014. Circadian clocks and inflammation: reciprocal regulation and shared mediators. Arch Immunol Ther Exp (Warsz). 62(4):303–318. 24687370 10.1007/s00005-014-0286-x

[bibr11-00220345251372506] Dawes C . 1972. Circadian rhythms in human salivary flow rate and composition. J Physiol. 220(3):529–545. 5016036 10.1113/jphysiol.1972.sp009721 PMC1331668

[bibr12-00220345251372506] Dawes C Ong BY . 1973. Circadian rhythms in the concentrations of protein and the main electrolytes in human unstimulated parotid saliva. Arch Oral Biol. 18(10):1233–1242. 4518743 10.1016/0003-9969(73)90035-6

[bibr13-00220345251372506] Druzd D Matveeva O Ince L Harrison U He W Schmal C Herzel H Tsang AH Kawakami N Leliavski A. 2017. Lymphocyte circadian clocks control lymph node trafficking and adaptive immune responses. Immunity. 46(1):120–132. 28087238 10.1016/j.immuni.2016.12.011 PMC5263259

[bibr14-00220345251372506] Flodby P Li C Liu Y Wang H Rieger ME Minoo P Crandall ED Ann DK Borok Z Zhou B. 2017. Cell-specific expression of aquaporin-5 (Aqp5) in alveolar epithelium is directed by GATA6/Sp1 via histone acetylation. Sci Rep. 7(1):3473. doi:10.1038/s41598-017-03152-7 28615712 PMC5471216

[bibr15-00220345251372506] Kaibori Y Tamoto S Okuda S Matsuo K Nakayama T Nagakubo D. 2024. CCL28: a promising biomarker for assessing salivary gland functionality and maintaining healthy oral environments. Biology (Basel). 13(3):147. doi:10.3390/biology13030147 38534417 PMC10968457

[bibr16-00220345251372506] Kalsbeek A Van der Spek R Lei J Endert E Buijs RM Fliers E. 2012. Circadian rhythms in the hypothalamo-pituitary-adrenal (HPA) axis. Mol Cell Endocrinol. 349(1):20–29. 21782883 10.1016/j.mce.2011.06.042

[bibr17-00220345251372506] Lee J Sul HJ Choi H Oh DH Shong M. 2022. Loss of thyroid gland circadian PER2 rhythmicity in aged mice and its potential association with thyroid cancer development. Cell Death Dis. 13(10):898. doi:10.1038/s41419-022-05342-2 36284088 PMC9596494

[bibr18-00220345251372506] Liu Y Weng X Wei M Yu S Ding Y Cheng B. 2022. Melatonin regulates the immune response and improves Sjögren’s syndrome–like symptoms in NOD/Ltj mice. Biochem Pharmacol. 201:115073. doi:10.1016/j.bcp.2022.115073 35525327

[bibr19-00220345251372506] Nater UM Rohleder N Schlotz W Ehlert U Kirschbaum C. 2007. Determinants of the diurnal course of salivary alpha-amylase. Psychoneuroendocrinology. 32(4):392–401. 17418498 10.1016/j.psyneuen.2007.02.007

[bibr20-00220345251372506] Out D Granger DA Sephton SE Segerstrom SC . 2013. Disentangling sources of individual differences in diurnal salivary α-amylase: reliability, stability and sensitivity to context. Psychoneuroendocrinology. 38(3):367–375. 22819683 10.1016/j.psyneuen.2012.06.013 PMC6988629

[bibr21-00220345251372506] Papagerakis S Said R Ketabat F Mahmood R Pundir M Lobanova L Guenther G Pannone G Lavender K McAlpin BR . 2022. When the clock ticks wrong with COVID-19. Clin Transl Med. 12(11):e949. doi:10.1002/ctm2.949 PMC9670202 36394205

[bibr22-00220345251372506] Qin B Wang J Yang Z Yang M Ma N Huang F Zhong R. 2015. Epidemiology of primary Sjögren’s syndrome: a systematic review and meta-analysis. Ann Rheum Dis. 74(11):1983–1989. 24938285 10.1136/annrheumdis-2014-205375

[bibr23-00220345251372506] Reppert SM Weaver DR . 2002. Coordination of circadian timing in mammals. Nature. 418(6901):935–941. 12198538 10.1038/nature00965

[bibr24-00220345251372506] Said R Lobanova L Papagerakis S Papagerakis P. 2020. Calcium sets the clock in ameloblasts. Front Physiol. 11:920. http://10.0.13.61/fphys.2020.00920 32848861 10.3389/fphys.2020.00920 PMC7411184

[bibr25-00220345251372506] Satou R Shibukawa Y Kimura M Sugihara N. 2019. Light conditions affect rhythmic expression of aquaporin 5 and anoctamin 1 in rat submandibular glands. Heliyon. 5(11):e02792. doi:10.1016/j.heliyon.2019.e02792 PMC6895735 31844723

[bibr26-00220345251372506] Strahler J Berndt C Kirschbaum C Rohleder N. 2010. Aging diurnal rhythms and chronic stress: distinct alteration of diurnal rhythmicity of salivary α-amylase and cortisol. Biol Psychol. 84(2):248–256. 20138206 10.1016/j.biopsycho.2010.01.019

[bibr27-00220345251372506] Uchida H Nakamura TJ Takasu NN Obana-Koshino A Ono H Todo T Sakai T Nakamura W. 2018. The central clock controls the daily rhythm of Aqp5 expression in salivary glands. J Physiol Sci. 68(4):377–385. 28484984 10.1007/s12576-017-0540-1 PMC10717580

[bibr28-00220345251372506] Ulrich-Lai YM Figueiredo HF Ostrander MM Choi DC Engeland WC Herman JP . 2006. Chronic stress induces adrenal hyperplasia and hypertrophy in a subregion-specific manner. Am J Physiol Metab. 291(5):E965–E973. doi:10.1152/ajpendo.00070.2006 16772325

[bibr29-00220345251372506] van der Horst GTJ Muijtjens M Kobayashi K Takano R Kanno S Takao M de Wit J Verkerk A Eker APM van Leenen D. 1999. Mammalian Cry1 and Cry2 are essential for maintenance of circadian rhythms. Nature. 398(6728):627–630. 10217146 10.1038/19323

[bibr30-00220345251372506] Wada M Orihara K Kamagata M Hama K Sasaki H Haraguchi A Miyakawa H Nakao A Shibata S. 2017. Circadian clock–dependent increase in salivary IgA secretion modulated by sympathetic receptor activation in mice. Sci Rep. 7(1):8802. doi:10.1038/s41598-017-09438-0 28821839 PMC5562870

[bibr31-00220345251372506] Wang Y Jiang W Chen H Zhou H Liu Z Liu Z Liu Z Zhou Y Zhou X Yu L. 2021. Sympathetic nervous system mediates cardiac remodeling after myocardial infarction in a circadian disruption model. Front Cardiovasc Med. 8:668387. doi:10.3389/fcvm.2021.668387 33842566 PMC8032890

[bibr32-00220345251372506] Zhang R Lahens NF Ballance HI Hughes ME Hogenesch JB . 2014. A circadian gene expression atlas in mammals: implications for biology and medicine. Proc Natl Acad Sci. 111(45):16219–16224. 25349387 10.1073/pnas.1408886111 PMC4234565

[bibr33-00220345251372506] Zheng L Ehardt L Mcalpin B About I Kim D Papagerakis S Papagerakis P. 2014. The tick tock of odontogenesis. Exp Cell Res. 325(2):83–89. 24582863 10.1016/j.yexcr.2014.02.007 PMC4072747

[bibr34-00220345251372506] Zheng L Seon YJ McHugh J Papagerakis S Papagerakis P. 2012. Clock genes show circadian rhythms in salivary glands. J Dent Res. 91(8):783–788. 22699207 10.1177/0022034512451450 PMC3398790

