# Supplementary material for: Effects of Circadian Misalignment on Young and Aging Salivary Glands
Source: J Dent Res. 2025 Oct 18;105(5):647–56. doi: 10.1177/00220345251372506 (PMC13068144; doi:10.1177/00220345251372506)
Supplement: Supplementary file 1 [file sj-docx-1-jdr-10.1177_00220345251372506.docx]

**TITLE: EFFECTS OF CIRCADIAN MISALIGNMENT ON YOUNG AND AGING SALIVARY GLANDS**

**Authors:** Helya Mortazavi ^1,2,3,4#^, Raed Said ^3,4#^, George S. Katselis ^5^, Paulos Chumala ^5^, Giuseppe Pannone ^6^, Silvana Papagerakis ^3,4,7*^, Petros Papagerakis ^1,3,4*^

1. Department of Anatomy, Physiology, and Pharmacology, College of Medicine, University of Saskatchewan, Saskatoon, Saskatchewan, Canada
2. Centre Hospitalier Universitaire de Québec Research Centre, Axe Maladies Infectieuses et Immunitaires, Université Laval, Quebec, Canada
3. ARThrite Research Center (Arthrite Research Treatment), Centre Hospitalier Universitaire de Québec Research Centre, Université Laval, Quebec, Canada
4. Interdisciplinary Center for Clinical and Applied Research and Entrepreneurship (I-CARE)/ Centre interdisciplinaire pour la recherche clinique et appliqué (CIRCA), Faculté de Médecine Dentaire, Université Laval, Quebec, Canada
5. College of Medicine, Core Mass Spectrometry Facility, Division of the Canadian Centre for Health and Safety in Agriculture, University of Saskatchewan, Saskatoon, Saskatchewan, Canada
6. Department of Clinical and Experimental Medicine, University of Foggia, Foggia, Italy
7. Department of Ophthalmology and ENT-Head and Neck Surgery, Faculty of Medicine, Université Laval, Québec, Canada

^#:^ equally contributing first authors

^*:^ corresponding authors

Address correspondence to: Silvana Papagerakis or Petros Papagerakis, 2420 Rue De La Terrasse Pavillon De Médecine Dentaire, Quebec City, Canada. Phone: (418) 656-5840; Email: silvana.papagerakis@fmd.ulaval.ca (SP) [petros.papagerakis@fmd.ulaval.ca](mailto:petros.papagerakis@fmd.ulaval.ca) (PP).

**MATERIALS & METHODS**

**Mice**

This study was approved by the University of Saskatchewan's Animal Research Ethics Board and adhered to the guidelines set forth by the Canadian Council on Animal Care for humane animal use (protocol no. 20200010). To minimize potential sex-related variations in circadian rhythms, exclusively female mice aged between 12 and 29 weeks were utilized. These mice were selected from five clock gene KO colonies and one wildtype (WT) colony serving as a control group and categorized as young (12 wks) and middle-aged or aging (29 wks) throughout the study. The colonies included are *Cry1KO* (JAX stock #016186), *Cry2KO* (JAX stock #016185) (Thresher et al. 1998), *Cry1&2DKO* (generated by crossing JAX stock #016186 and JAX stock #016185), *Per2KO* and *Bmal1KO* (obtained from Dr. Willemijntje and Dr. A. Hoogerwerf, Department of Internal Medicine, University of Michigan (Bunger et al., 200; Bae et al. 2001; Hoogerwerf et al. 2010)). Each of these models carries distinct systemic phenotypes. Cry1KO and Cry2KO mice display altered circadian periodicity but generally retain rhythmicity: Cry1KO mice have a shortened circadian period, while Cry2KO mice have a lengthened period. Both are viable, fertile, and exhibit only mild metabolic or behavioral changes under standard conditions (Thresher et al., 1998). In contrast, Cry1&2 double knockout (Cry1&2DKO) and Bmal1KO mice are completely arrhythmic in constant darkness, with profound systemic effects. Cry1&2DKO mice are viable but display metabolic syndrome features, immune dysregulation, and increased tumor susceptibility (van der Horst et al., 1999). Bmal1KO mice exhibit the most severe systemic phenotype, including complete loss of circadian rhythmicity, premature aging, infertility, metabolic syndrome, and reduced lifespan (Bunger et al., 2000; Kondratov et al., 2006). Per2KO mice typically show a shortened free-running period, but many—especially females—become arrhythmic after prolonged constant darkness, with increased cancer risk and metabolic disturbances (Bae et al., 2001; Zheng et al., 2001).

To identify and select mutant mice from each colony, tail clips were obtained from anesthetized mice. Genomic DNA was then extracted using the REDExtract-N-Amp Tissue PCR Kit (Sigma-Aldrich, USA) according to the manufacturer's instructions. The primer protocols utilized for genotyping assays are listed in table 1. The study was designed with a sample size of four mice per group across the two distinct age categories, based on previous research on the lacrimal gland phenotype (Stefanski et al. 2017). The mice were housed in the Lab Animal Services Unit (LASU) at the University of Saskatchewan. They were fed ad libitum and maintained on a normal light-dark cycle. Environmental enrichment was provided to enhance their welfare and stimulate natural behaviors. This setup aimed to ensure the mice's physical and behavioral needs were met, aligning with best practices for laboratory animal care. To ensure the humane treatment of the mice, a Humane Intervention Point (HIP) checklist was designed and implemented with the LASU veterinary staff. This checklist monitored the mice's health status and provided guidelines for intervention based on their condition. The HIP list included the parameters for monitoring animal health (based on physical appearance, water/food intake, body weight, natural and provoked behaviour) to identify any signs of distress or illness, a detailed scoring system based on the parameters and outlined actions to be taken if certain scores were met. These actions included increasing the frequency of monitoring, administering analgesics or supportive therapy, and notifying the principal investigator or veterinary staff. In cases where the mice's condition exceeded a certain severity threshold, euthanasia was performed to prevent unnecessary suffering.

**Samples Collection**

Collections of saliva and blood were scheduled at two distinct times: 9:00-10:00 am (early rest period) and 9:00-10:00 pm (early active period), to account for circadian variations. For saliva collection, mice were first weighed and then placed in a clean cage without access to food or water for one hour. Saliva production was then stimulated through a subcutaneous injection of pilocarpine (1μg/g body weight), a parasympathetic agonist, in sterile saline. The protocol for pilocarpine preparation was previously described (Varghese et al. 2018). Saliva was collected in a microcentrifuge tube from the oral cavity using a micropipette and sterile filtered tips for 15 minutes, followed by centrifugation at 4°C. The volume of saliva collected was measured, and the salivary flow rate was calculated as μL per 100 grams of body weight (Salam et al. 2004). Blood samples were obtained by extracting a volume equivalent to 0.07% of the mouse's body weight from the facial vein. This was achieved using a lancet and a BD Microtainer™ Capillary blood collector with microgard™ closure (Becton, Dickinson and Company (BD), USA). All collected samples were immediately stored at -80°C for future analysis. Salivary glands, including the submandibular, parotid (PSG), and sublingual glands (SLGs), were harvested from the groups. The glands were dissected, and gross morphological comparisons were conducted between the groups. For histological analysis, half of each gland was fixed in 10% neutral buffered formalin, embedded in paraffin, and sectioned. The other half was preserved in RNA later (Qiagen, USA) for subsequent molecular analysis. The paraffin-embedded blocks were sectioned at 4.5 μM thickness, air-dried at 37°C for 24 hours, and then stored at 4°C until staining.

**Histological Staining and Immunohistochemistry**

Hematoxylin & Eosin (H&E) Staining Protocol: Slides were sequentially immersed in 100% xylene for 3 minutes, repeated twice, followed by a 1-minute immersion in a 50:50 mixture of xylene and 100% ethanol. This was followed by a 1-minute immersion in 100% ethanol, repeated once. Subsequently, slides were transferred to 95% ethanol for 1 minute and then rinsed with distilled water for 1 minute. The slides were then dipped into Harris hematoxylin (Thermo Fisher Scientific, Waltham, Massachusetts, USA) for 2 minutes, followed by thorough rinsing with tap water until the water ran clear. Acid alcohol (0.5% HCl in 95% ethanol) was used for a brief 1-second immersion, repeated twice until the slides turned pink, followed by three rinses with tap water. After this, slides were immersed in saturated aqueous lithium carbonate for 5 seconds, and a 3-minute rinse with running tap water and distilled water. Next, the slides were treated with eosin Y (Thermo Fisher Scientific, Waltham, Massachusetts, USA) for 1 minute and 15 seconds. Post-eosin Y treatment, slides were consecutively submerged in 70% ethanol for 1 minute, 95% ethanol for 1 minute, and 100% ethanol for 1 minute, repeated once. Subsequently, slides were placed in a 50:50 mixture of xylene and 100% ethanol for 1 minute, followed by 100% xylene for 1 minute, repeated thrice. Finally, coverslips were applied using Surgipath MM24 mounting media (Leica Biosystems, Wetzlar, Germany). *Analysis:* To assess the effects of circadian misalignment and aging on salivary epithelial and stromal cell size and proliferation, we measured the size of 100 sequential acini in the submandibular glands per mouse and we measured the parenchyma to stroma ratio in the submandibular glands using sections cut at the same level (i.e. the submandibular glands were sectioned in their ventro-dorsal center).

Focus score: Focal lymphocytic sialadenitis (FLS) was assessed according to the modified Chisholm and Mason scoring system, in which a focus is defined as an aggregate of ≥50 mononuclear cells (primarily lymphocytes) per 4 mm² of glandular tissue. For each gland, the total number of foci was counted across all visible lobules, and the total glandular area (excluding adipose and connective tissue) was quantified using ImageJ (NIH). The focus score was calculated as follows:

{Focus Score} = {Number of foci}\Glandular area (mm^2) * 4

Only periductal or perivascular lymphocytic infiltrates located within acinar regions were considered. Sections with tissue artifacts or insufficient glandular area (<1 mm²) were excluded from analysis.

Periodic Acid-Schiff and Alcian Blue Staining Protocol (PAS/AB): We used the histochemical markers Periodic Acid-Schiff and Alcian Blue (pH 2.5) (#38016SS4A, Leica Biosystems, Wetzlar, Germany) (PAS/AB) to stain mucus acinar cells. The staining procedure was as follows: 1. Tissue sections were deparaffinized in xylene. 2. Sections were immersed in a 3% aqueous acetic acid solution for 3 minutes. 3. Slides were then placed in Alcian Blue solution for 30 minutes at room temperature. 4. Sections were washed in running tap water for 10 minutes. 5. The slides were transferred to a 0.5% aqueous periodic acid solution for 10 minutes. 6. Post-washing, the sections were immersed in Schiff reagent for 20 minutes. 7. Slides were washed again in running tap water and then dehydrated by graded series of ethyl alcohol. This staining protocol resulted in distinct coloration of various cellular components: acidic epithelial cells exhibited blue coloration, neutral epithelial mucin and glycogen appeared magenta/red, and mixed mucins (neutral and acidic) appear purple to blue (Bancroft and Gamble 2008).

Whole-section images were captured 40× objective and identical light and exposure settings across all samples. For consistency and to minimize sampling bias, three random, non-overlapping regions were selected of each gland section. ROIs were drawn manually in Fiji/ImageJ to encompass representative glandular epithelial tissue while excluding large blood vessels, fat, and connective tissue. Digital images were analyzed using Fiji/ImageJ. Quantification was performed using the Colour Deconvolution plugin in Fiji, which implements the Ruifrok and Johnston method. This approach separates the image into stain-specific channels based on the known absorption characteristics of each dye. For AB and PAS stains, the built-in vectors “Alcian blue & hematoxylin” and “Haematoxylin and Periodic Acid-Schiff (PAS)” were selected as appropriate. If necessary, custom vectors were determined by sampling representative stained regions using rectangular ROIs, following plugin instructions. The plugin generated separate grayscale images for each stain, where pixel intensity corresponded to the optical density of the specific stain. For each deconvolved channel (AB and PAS), a consistent threshold was applied to segment positive staining from background. Threshold values were established empirically on representative images and then uniformly applied to all samples. The “Analyze > Set Measurements” tool was configured to record area, mean gray value, and area fraction. The “Analyze > Measure” function was used to quantify the stained area and intensity within each section. For each ROI, the mean gray value (MGV) was calculated using Analyze > Measure. Lower MGV indicates higher stain density. To allow direct comparison, MGVs were inverted (255 – MGV) to yield staining intensity scores. Mean staining intensity for Alcian Blue and PAS was averaged across three ROIs per sample. For each stain, data were analyzed using two-way ANOVA (factors: genotype, age) with Tukey’s post hoc test to assess pairwise differences. Significance was set at p < 0.05. Graphs were generated using GraphPad Prism.

Immunohistochemistry: Immunohistochemistry (IHC) was performed using the polymeric horseradish peroxidase (HRP)-linker antibody conjugate technique in conjunction with the Proteinase K Antigen Retrieval Protocol (# RE7160-K, Leica Biosystems, Wetzlar, Germany). The procedure began with slide preparation, which included baking, deparaffinization, and blocking of endogenous peroxidase activity. The tissue sections were subsequently incubated with the primary antibody (rabbit anti-CD3 antibody, #14-0031-82, Thermo Fisher, Waltham, Massachusetts, USA) or (rabbit anti-alpha amylase, 15845-1-AP, Thermo Fisher, USA), followed by probing with a peroxidase-labeled secondary antibody. Staining was facilitated using a peroxidase complex, with DAB chromogen (ab64238, Abcam, Cambridge, United Kingdom) serving as the substrate. After staining, the slides were toned, counterstained with hematoxylin, and cover slipped. Stained slides were assessed using the Aperio Virtual Microscopy System (Leica Biosystems, Wetzlar, Germany) to evaluate histological differences. Semi-quantitative IHC analysis to determine CD3-positive cells was conducted utilizing ImageJ (https://imagej.nih.gov/ij/index.html), as previously described (Schneider et al. 2012).

Immunofluorescence (IF): we used the primary antibodies rabbit anti-Aqp5 (1:100) (#A5-99403, Invitrogen, USA) and the secondary antibodies Alexa Fluor 477 goat anti-rabbit (Invitrogen, Invitrogen, USA), Subsequently, all sections were counterstained by DAPI and mounted with Prolong Gold antifade reagent (Invitrogen, USA).

**Quantitative Real-Time Polymerase Chain Reaction (qRT-PCR)**

Total ribonucleic acid (RNA) extraction was performed separately for the parotid and submandibular glands using the RNeasy Minikit (#74104, Qiagen, Hilden, Germany). Following complementary DNA (cDNA) synthesis (4368814, Applied Biosystems, Waltham, Massachusetts, USA), qRT-PCR analysis was conducted using the Applied Biosystems instrument to evaluate the expression levels of specific genes associated with salivary gland function. The genes analyzed included Interleukin-17 alpha (*IL17A*), Sjögren's syndrome A antigen (SSA/Ro60), tumor necrosis factor-alpha (*TNFa*), Aquaporin 5 (*Aqp5*), Amylase (*Amy*), and *Mucin 19*. The 2^^-ΔΔCt^ method was employed for the relative quantification of gene expression levels. Beta-2 microglobulin (*B2M*) was selected as a reference gene, as it is unaffected by circadian regulation. Primers with melting temperatures between 59–60 °C were designed through GenScript, spanning introns of the target genes, with amplicon sizes ranging from 100-150 bp. Primer sequences used are listed in table 1.

| **Target** | **Forward primer** | **Reverse primer** |
| --- | --- | --- |
| Cryptochrome 1 | TACAGGCAGCAGTGAGGAAG - common | GGCCATGTGGCTATCTGTTC - wildtype  ATCGCCTTCTATCGCCTTCT - mutant |
| Cryptochrome 2 | AAGACAGGCTTCCCTTGGAT - common | GGAGCAGCTCGTCAAATACC - wildtype  GGATGATCTGGACGAAGAGC - mutant |
| Brain and Muscle ARNT-Like 1 | CCACCAAGCCCAGCAACTCA - common | ATTCGGCCCCCTATCTTCTG - wildtype  TCGCCTTCTATCGCCTTCTT -mutant |
| Period 2 Circadian Regulator | AGAACTTGTTGCTCCTGCTT - common | GGAAGCTTGTAAGGGGTGGT- wildtype  TGC CCCAAAGGCCTACCA GCTTCC- mutant |
| Interleukin-17 alpha | CAAACACTGAGGCCAAGGAC | ACGTGGAACGGTTGAGGTAG |
| Sjögren's syndrome A | AGACGGCAGAGGATGTGAAG | TGAAGAGGTGGGTCGGAATG |
| Tumour necrosis factor-alpha | TCTACTCCCAGGTTCTCTTC | AGACTCCTCCCAGGTATATG |
| Aquaporin5 | TCACTGGGTCTTCTGGGTAG | CAGCTCGATGGTCTTCTTCC |
| Amylase | CAGCACTTGTGGCAATGACTGG | GCAAAAGGCTGACCATTGACGAC |
| Mucin19 | CTGGGTCTGGAAGTAGAAGTA | TCTAAGCCACAGAAGGAGAT |
| Beta-2-Microglobulin | GTCTTTCTGGTGCTTGTCTC | GTATGTTCGGCTTCCCATTC |

**Appendix Table1:** PCR and qPCR primers sequences. Sequences are presented in the 5’ to 3’ orientation.

**Statistical Analysis**

All statistical analyses were performed by R Studio and SPSS version 29. The Kruskal-Wallis test was selected to compare differences in mandibular gland area size, saliva production (morning and night), and the expression of various immunological *(IL-17, SSA, TNFα*) and functional markers (*Aqp5, Mucin19, Amy*) across different genotypes and age groups. To further investigate the effect of aging on gene expression, the Mann-Whitney U-test was applied, focusing on comparing two independent groups to assess aging effects. A Chi-squared test was selected to assess the differences in categorical data related to mandibular gland area size, saliva production, and the expression of immunological/physiological markers across merged age groups and various genotypes.

**QUANTITATIVE PROTEOMIC ANALYSIS OF MOUSE BLOOD SAMPLES**

**Methods**

Blood samples from aging mice across all colonies were utilized for the assessment of protein quantities. It's important to highlight that attempts were made initially to prepare saliva samples for proteomic analysis as previously outlined (Marin et al. 2023). Unfortunately, the protein concentrations in these saliva samples were notably low, rendering further quantitative proteomics analysis in saliva samples unfeasible. Depletion of high-abundance proteins: Blood samples were processed to quantify low-abundance proteins. The multiple affinity removal system (MARS)-3 depletion spin cartridge (Agilent Technologies, Santa Clara, California, USA) was used following the manufacturer's protocol to deplete high-abundance proteins, retaining only the low-abundance fraction for mass spectrometry analysis. Protein concentrations were analyzed using the Pierce BCA Protein Assay Kit (Thermo Scientific, Waltham, Massachusetts, USA). Sample preparation for liquid chromatography-tandem mass spectrometry (LC-MS/MS): Samples underwent trypsin digestion as described previously. Briefly, proteins were denatured with trifluoroethanol (Fisher Scientific, Waltham, Massachusetts, USA), reduced with dithiothreitol (MP Biomedicals, Santa Ana, California, USA), and alkylated with iodoacetamide (Fisher Scientific, Waltham, Massachusetts, USA). Contaminants were removed by acetone precipitation and protein pellets were resuspended in ammonium bicarbonate for overnight digestion with Pierce Trypsin Protease MS-Grade (Thermo Scientific, Waltham, Massachusetts, USA). Digests were desalted prior to LC-MS/MS analysis. LC-MS/MS analysis and protein identification: Digested samples were analyzed in duplicate on an Agilent 6550 iFunnel Q-TOF coupled to an Agilent 1260 LC system using a Chip Cube interface. Peptides were separated on a Polaris-HR-Chip 3C18 column with a water/acetonitrile gradient containing 0.1% formic acid. MS/MS data were acquired in positive ion mode over m/z 250-1700 at 8 spectra/s, with the top 20 precursors selected for fragmentation using active exclusion (0.25 min). Tandem mass spectra were searched against the UniProtKB Swiss-Prot database using Spectrum Mill (Agilent) for protein identification (Nabeta et al. 2022). Data analysis: Principal component analysis (PCA) removed outliers prior to statistical testing. Relative protein abundances were compared using parametric (t-tests, ANOVA) and non-parametric tests (Kruskal-Wallis, Jonckheere-Terpstra) based on data normality, with Benjamini-Hochberg correction for multiple testing. Pathway and functional analyses utilized curated databases (Reactome, PID, KEGG, BIOCARTA) with Fisher's exact test and the STRING, BIOGRID and WebGestalt tools. Partial least squares discriminant analysis (PLS) identified proteins linked to clock gene disruption, with variable importance in projection scores (VIP) > 0.5 considered significant contributors. The final biomarker proteins were validated against raw intensities and fragmentation patterns (Karimpour-Fard et al. 2015).

**RESULTS (Appendix Table 2)**

Serum proteomic signatures revealed disruption of core circadian regulators resulted in significant proteomic pathway alterations and potential immune-related biomarkers. *Cry1KO* mice exhibited upregulation in pathways such as glycolysis/gluconeogenesis, MAPK signaling, chemokine signaling, AMPK signaling, and regulation of the actin cytoskeleton. These changes suggest alterations in cellular metabolism, signaling, and cytoskeletal dynamics associated with the Cry1 gene. Out of four protein features *Aldehyde dehydrogenase (Aldh3a1), filamin-A (Flna), Platelet factor 4 (Pf4), Ras-related protein (Rab10),* and *Myosin-9(Myh9*), all with VIP scores greater than one, *Pf4, Rab10,* and *Myh9* were significantly enriched in immune system processes, indicating their critical role in the observed biological changes. *Cry2KO* mice showed upregulation of the PI3K-Akt signaling pathway, protein-lysine N-methyltransferase, and tyrosine/phenylalanine metabolism pathways, along with downregulation of complement and coagulation cascades. This pattern reflects metabolic, immune, and coagulation-related changes linked to the absence of the Cry2 gene. Notably, proteins *14-3-3 protein zeta/delta (Ywhab), Clusterin (Clu), Elongation factor 2 (Eef2),* and *Macrophage migration inhibitory factor (Mif),* all with VIP scores greater than one were significantly enriched in immune system processes.

*DKOCry* mice demonstrated upregulation in pathways related to lysosomal trafficking, Ras signaling, neurodegeneration, and complement cascades. These findings highlight biomarkers associated with neurodegenerative processes, immune modulation, and cellular metabolism. Among the five protein features, *Lysosomal trafficking regulator (Lyst) ,* and *Ral GTPase activating protein, alpha subunit 2 (Ralgapa2*) had VIP scores greater than one, with *Lyst* significantly enriched in immune system processes. *Per2KO* mice had upregulated pathways linked to immune responses, such as neutrophil extracellular trap formation and TGF-beta signaling, alongside downregulated pentose phosphate, complement, and coagulation pathways. These alterations suggest biomarkers of immune activation, inflammation, and metabolic and coagulation dysregulation. *Platelet-derived growth factor receptor alpha (Pdgfra)* and *Fibrinogen alpha chain (Fga)*, both with VIP scores greater than one, were significantly enriched in immune system processes. *Bmal1KO* mice showed upregulation in pathways involving platelet activation, Rap1 signaling, and apoptosis. These changes indicate potential biomarkers related to cellular signaling, platelet function, and apoptotic processes. Proteins *Rho guanine nucleotide exchange factor 9 (Arhgef9),* and *Actin (Actb)*, with VIP scores greater than one, were significantly enriched in immune system processes.

B

C

| Colony | Protein Gene Symbol | Variable Importance in Projection | Fold Change | Protein name | Key Pathways |
| --- | --- | --- | --- | --- | --- |
| *Cry1 KO* | Aldh3a1 | 1.317 | 24.49 | Aldehyde dehydrogenase | Glycolysis / Gluconeogenesis |
|  | Flna | 1.292 | 28.24 | Filamin-A | MAPK signalling pathway |
|  | Pf4 | 1.287 | 6.88 | Platelet factor 4 | Chemokine signaling pathway, Cytokine-cytokine receptor interaction |
|  | Rab10 | 1.111 | 24.53 | Ras-related protein Rab-10 | AMPK signalling pathway, Endocytosis |
|  | Myh9 | 1.027 | 25.77 | Myosin-9 | Regulation of actin cytoskeleton |
| *Cry2 KO* | Ywhaz | 1.446 | 19.94 | 14-3-3 protein zeta/delta | PI3K-Akt signalling pathway |
|  | Clu | 1.204 | -2.93 | Clusterin | Complement and coagulation cascades |
|  | Eef2 | 1.169 | 24.48 | Elongation factor 2 | Protein-lysine N-methyltransferase |
|  | Mif | 1.119 | 2.68 | Macrophage migration inhibitory factor | Tyrosine metabolism, Phenylalanine metabolism |
| *DKOCry* | Lyst | 5.002 | 2.83 | Lysosomal trafficking regulator | Lysosomal-trafficking |
|  | Ralgapa2 | 2.968 | 1.79 | Ral gtpase activating protein | Ras signaling pathway |
|  | Kif5b | 0.35 | 9.79 | Kinesin-1 | Pathways of neurodegeneration - multiple diseases |
|  | Cfb | 0.317 | 3.05 | Complement factor B | Complement and coagulation cascades |
|  | Setd3 | 0.35 | 13.81 | Histone-lysine N-methyltransferase | Protein-histidine N-methyltransferase |
| *Per2 KO* | Fpr-rs6 | 0.496 | 25.51 | Formyl peptide receptor-related sequence 6 | Neutrophil extracellular trap formation |
|  | Dcn | 0.503 | 24.75 | Decorin | TGF-beta signaling pathway |
|  | Pdgfra | 2.064 | 31.90 | Platelet-derived growth factor receptor alpha | Rap1, MAPK, PI3K-Akt, JAK-STAT, and Ras signalling pathways |
|  | G6pdx | 0.499 | 1.95 | Glucose-6-phosphate 1-dehydrogenase X | Pentose phosphate pathway |
|  | Fga | 1.606 | 1.55 | Fibrinogen alpha chain | Complement and coagulation cascades, Platelet activation |
| *Bmal1 KO* | Arhgef9 | 1.255 | 31.71 | Rho guanine nucleotide exchange factor 9 | Rho GTPase-associated proteins |
|  | Ppib | 0.49 | 24.41 | Peptidyl-prolyl cis-trans isomerase b |  |
|  | Actb | 2.403 | 24.73 | Actin, cytoplasmic 1 | Platelet activation, Rap1 signalling pathway, Apoptosis |

**Appendix Table 2: Summary of differentially expressed proteins in the serum of aging clock knockout mice when compared to wildtypes**

**Discussion**

Our serum proteomic profiling unveiled several altered pathways reflective of immune responses in the different KO models. Of note, the Lysosomal trafficking regulator (LYST) and Ral gtpase activating protein (RALGAPA2) proteins were significantly upregulated in *Cry1/2 DKO* mice serum (i.e. the most SS-like model). LYST is a well-known immune modulator and LYST mutations result in Chédiak-Higashi syndrome(Turner et al. 2024), an autosomal recessive immunodeficiency, but specific research linking it directly to SS is not well-documented. Another protein of interest observed was the macrophage migration inhibitory factor (MIF) which was significantly upregulated in *Cry2 KO*. MIF is a pro-inflammatory cytokine involved in various autoimmune diseases, including SS(Harris et al. 2019). Indeed, studies have shown that MIF plays a significant role in the pathogenesis of SS by promoting inflammatory responses and potentially contributing to glandular dysfunction and destruction (Rizo et al. 2015)(Xiang et al. 2023).

**LIMITATIONS**

While our study provides valuable insights, it's important to acknowledge its limitations: 1. The use of global knockout models makes it challenging to distinguish between local and systemic effects of circadian disruption on salivary glands. 2. Although we observed age-specific changes, our study was limited to two time points. A more comprehensive longitudinal study could provide deeper insights into age-related progression of salivary gland dysfunction. 3. Our study did not extensively explore potential sex differences in circadian regulation of salivary gland function, which could be significant given the higher prevalence of SS in females.

**REFERENCES**

Bae, Kiho, Xiaowei Jin, Elizabeth S. Maywood, Michael H. Hastings, Steven M. Reppert, and David R. Weaver. "Differential functions of mPer1, mPer2, and mPer3 in the SCN circadian clock." Neuron 30, no. 2 (2001): 525-536.

Bancroft JD, Gamble M. 2008. Theory and practice of histological techniques.

Bunger, Maureen K., Lisa D. Wilsbacher, Susan M. Moran, Cynthia Clendenin, Laurel A. Radcliffe, John B. Hogenesch, M. Celeste Simon, Joseph S. Takahashi, and Christopher A. Bradfield. "Mop3 is an essential component of the master circadian pacemaker in mammals." Cell 103, no. 7 (2000): 1009-1017.

Harris J, VanPatten S, Deen NS, Al-Abed Y, Morand EF. 2019. Rediscovering MIF: new tricks for an old cytokine. Trends Immunol. 40(5):447–462.

Hoogerwerf WA, Shahinian VB, Cornélissen G, Halberg F, Bostwick J, Timm J, Bartell PA, Cassone VM. 2010. Rhythmic changes in colonic motility are regulated by period genes. American Journal of Physiology-Gastrointestinal and Liver Physiology. 298(2):G143-G150.

Karimpour-Fard A, Elaine Epperson L, Hunter LE. 2015. A survey of computational tools for downstream analysis of proteomic and other omic datasets. Human Genomics. 9(1).

Marin LM, Katselis GS, Chumala P, Sanche S, Julseth L, Penz E, Skomro R, Siqueira WL. 2023. Identification of sars-cov-2 biomarkers in saliva by transcriptomic and proteomics analysis. Clinical Proteomics. 20(1):30.

Nabeta R, Katselis GS, Chumala P, Dickinson R, Fernandez NJ, Meachem MD. 2022. Identification of potential plasma protein biomarkers for feline pancreatic carcinoma by liquid chromatography tandem mass spectrometry. Veterinary and Comparative Oncology. 20(3):720-731.

Rizo VD, Robledo NSF, Vazquez JDM, Guerrero EP, Trujillo X, Lopez LG, Nava JIG. 2015. FRI0430 Association Between Circulating Macrophage Migration Inhibitory Factor (MIF) with Adipokines Levels, Auto-Antibodies and Pro-Inflammatory Cytokines in Lupus Nephritis. Ann Rheum Dis. 74:582–583.

Salam MA, Matsumoto N, Matin K, Tsuha Y, Nakao R, Hanada N, Senpuku H. 2004. Establishment of an animal model using recombinant nod. B10. D2 mice to study initial adhesion of oral streptococci. Clinical and Vaccine Immunology. 11(2):379-386.

Schneider CA, Rasband WS, Eliceiri KW. 2012. Nih image to imagej: 25 years of image analysis. Nature Methods 2012 9:7. 9(7):671-675.

Stefanski A-L, Tomiak C, Pleyer U, Dietrich T, Burmester GR, Dörner T. 2017. The diagnosis and treatment of sjögren’s syndrome. Deutsches Ärzteblatt International. 114(20):354.

Thresher, Randy J., Martha Hotz Vitaterna, Yasuhide Miyamoto, Aleksey Kazantsev, David S. Hsu, Claude Petit, Christopher P. Selby et al. "Role of mouse cryptochrome blue-light photoreceptor in circadian photoresponses." Science 282, no. 5393 (1998): 1490-1494.

Turner ME, Che J, Mirhaidari GJM, Kennedy CC, Blum KM, Rajesh S, Zbinden JC, Breuer CK, Best CA, Barker JC. 2024. The lysosomal trafficking regulator “LYST”: an 80-year traffic jam. Front Immunol. 15:1404846.

Varghese JJ, Schmale IL, Hansen ME, Newlands SD, Benoit DS, Ovitt CE. Murine salivary functional assessment via pilocarpine stimulation following fractionated radiation. Journal of visualized experiments: JoVE. 2018 May 4(135):57522.

Xiang Y, Zhang M, Jiang D, Su Q, Shi J. 2023. The role of inflammation in autoimmune disease: a therapeutic target. Front Immunol. 14:1267091.
